# RNF182 induces p65 ubiquitination to affect PDL1 transcription and suppress immune evasion in lung adenocarcinoma

**DOI:** 10.1002/iid3.864

**Published:** 2023-05-22

**Authors:** Xingdu Zeng, Xiaoyuan Tang, Xingxiang Chen, Huilan Wen

**Affiliations:** ^1^ Department of Respiratory Medicine The First Affiliated Hospital of Gannan Medical University Ganzhou Jiangxi People's Republic of China

**Keywords:** E3 ubiquitin ligase, immune evasion, NF‐kB p65, PDL1, RNF182

## Abstract

**Background:**

The RING finger (RNF) proteins are a large group of ubiquitin ligases whose aberrant expression is often associated with disease progression. This study examines the function of RNF protein 182 (RNF182) in lung adenocarcinoma (LUAD) cells and its impact on p65 and programmed death ligand 1 (PDL1) regulation.

**Methods:**

Expression of RNF182, p65, and PDL1 in LUAD tissues and cells was measured using immunohistochemistry, reverse transcription quantitative polymerase chain reaction (RT‐qPCR), and/or western blot (WB) assays. LUAD cells were induced to overexpress RNF182 and p65, followed by cell counting kit‐8, colony formation, Transwell, and flow cytometry assays to evaluate the cells’ malignant phenotype. Coimmunoprecipitation and WB assays were used to verify RNF182's effect on p65 ubiquitination. Chromatin immunoprecipitation‐qPCR and luciferase assays were used to analyze p65's transcriptional regulation of PDL1. Coculture of LUAD with CD8^+^ cytotoxic T cells was performed to detect lactate dehydrogenase release and interferon‐γ and interleukin‐2 concentrations. LUAD cells were implanted in mice to analyze tumorigenicity.

**Results:**

RNF182 was poorly expressed, while p65 and PDL1 were highly expressed in LUAD tissues and cells. RNF182 overexpression suppressed the malignant properties of LUAD cells, and it promoted p65 ubiquitination and protein degradation. p65 activated PDL1 transcription. Overexpression of RNF182 suppressed the PDL1 expression, increased the cytotoxicity in LUAD cells cocultured with CD8^+^ T cells, and suppressed the tumorigenesis of cancer cells in vivo. However, these tumor‐suppressive effects of RNF182 on LUAD cells were blocked by p65 restoration.

**Conclusion:**

This research demonstrates that RNF182 induces p65 ubiquitination to suppress PDL1 transcription and immunosuppression in LUAD.

## Graphical abstract

This work defines a novel tumor‐suppressive E3 ubiquitin ligase RING finger protein 182 (RNF182) which induces ubiquitination and degradation of p65 and consequently suppresses the transcription of programmed death ligand 1, therefore, alleviating immunosuppression and cancer development. These findings may offer new ideas that RNF182 may be used as a strategy to help reduce immune escape and limit tumor growth in lung adenocarcinoma.

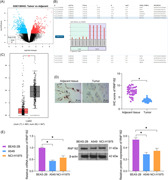

## 1 INTRODUCTION

Lung cancer stands for the second commonest cancer (11.4%) and the unassailable leading cause of cancer‐related mortality (18%) worldwide in 2020 among all cancer types.^1^ Lung adenocarcinoma (LUAD) represents the primary subtype that accounts for over 40% of all cases.^2^ Despite improvements in therapeutic options, such as noninvasive surgical resection, immunotherapy, and chemoradiotherapy, treatment outcomes remain unsatisfactory.^3^, ^4^

Ubiquitination is an important posttranslational process that plays a role in a variety of pathogenesis, including carcinogenesis.^5^ The E3 ubiquitin ligases exert critical functions in inducing protein degradation via the 26S proteasome by ubiquitin‐tagging the proteins for destruction.^6^ RING finger (RNF) proteins are a large group of ubiquitin ligases.^7^ In this study, we obtained RNF182 as a differentially expressed gene (DEG) in LUAD by analyzing the Gene Expression Omnibus (GEO) data set GSE136043. RNF182 is mainly expressed in nervous system tissues, where is abnormal expression has been associated with neuronal apoptosis and neural impairments.^7^ However, little is known about its expression in the lungs or its relevance to carcinogenesis.

RNF182 has been reported to modulate the ubiquitination and degradation of p65.^8^ p65, also known as RELA, is one of the major subunits of the nuclear factor kappa B (NF‐κB) pathway with the ability to induce target gene transcription and promote tumor development.^9^ Elevated NF‐κB p65 expression has been detected in lung cancer as well,^10^, ^11^ and p65 upregulation has been linked to poor prognosis.^12^ Moreover, p65 has been demonstrated to activate the transcription of programmed death ligand 1 (PDL1), a core immune checkpoint receptor that directly leads to immune evasion and creates an immunosuppressive condition for lung cancer development.^13^ Therefore, we sought to investigate whether RNF182 is linked to the immune response and malignant development of LUAD by regulating the p65/PDL1 axis. This study was performed to validate the interactions and roles of RNF182 and the p65/PDL1 axis in LUAD progression using two LUAD cell lines A549 and NCI‐H1975.

## 2 MATERIALS AND METHODS

### 2.1 Collection of clinical samples

The use of human samples was approved by the Ethics Committee of the First Affiliated Hospital of Gannan Medical University (Approval No. LLSC‐2019032710). All procedures were in accordance with the principles of the *Declaration of Helsinki*, and informed consent was received from all patients. Tumor and the adjacent noninvolved para‐tumorous tissues from 47 patients who underwent surgery at the First Affiliated Hospital of Gannan Medical University were collected. None of the patients had not receive any anticancer therapy before surgery. The tissue samples were stored at −80°C.

### 2.2 Immunohistochemistry (IHC)

The collected tissue samples were fixed and paraffined for sectioning. The sections were dewaxed, heated in citrate buffer, blocked in 3% H_2_O_2_ for 30 min, and then reacted with the antibodies of RNF182 (1:200, PA5‐52925; Thermo Fisher Scientific), p65 (1:500, 8242; Cell Signaling Technology [CST]), and PDL1 (1:500, ab205921; Abcam Inc.) overnight at 4°C, and then with immunoglobulin G (IgG) (horseradish peroxidase [HRP]) (1:1000, ab6721; Abcam) for 45 min at 22–25°C. Afterward, the sections were stained with 3,3′‐diaminobenzidine and counterstained with hematoxylin, followed by neutral balsam sealing for microscopy analysis.

### 2.3 Cells

Human normal lung epithelial cells BEAS‐2B (CL‐0496), human LUAD cells A549 (CL‐0016) and NCI‐H1975 (CL‐0298), and 293T cells (CL‐0005) were procured from Procell Life Science & Technology. BEAS‐2B and 293T cells were incubated in Dulbecco's modified Eagle's medium, A549 cells in F12K medium, and NCI‐H1975 cells in Roswell Park Memorial Institute‐1640. All media were supplemented with 10% fetal bovine serum (FBS), 1% antibiotics, and 1% glutamine. The culture condition for all cells were 37°C with 5% CO_2_.

The overexpression plasmids of RNF182 and p65 (oe‐RNF182 and oe‐p65), the negative control (NC) plasmids, and the lentivirus vector for packaging were procured from VectorBuilder Inc. The lentivirus of oe‐RNF182 was puromycin‐resistant, while that of oe‐p65 was hygromycin‐resistant. The cells were treated with the lentivirus vectors until reaching 60% confluence. After 48 h,^14^ stably transfected cells were screened by the addition of corresponding antibiotics.

### 2.4 Reverse transcription quantitative polymerase chain reaction (RT‐qPCR)

TRIzol reagent was used for RNA extraction, and the cDNA for qPCR analysis was obtained using the PrimeScript RT Reagent Kit with gDNA Eraser (RR047A; TaKaRa Holdings Inc.). The following primers are used: RNF182 (F): 5ʹ‐GTCCTCCAACTGCCTGGTCATA‐3ʹ, RNF182 (R) 5ʹ‐GTGGTGACAGAGTCGAAACTCG‐3ʹ; PDL1 (F): 5ʹ‐TGCCGACTACAAGCGAATTACTG‐3ʹ, PDL1 (R): 5ʹ‐CTGCTTGTCCAGATGACTTCGG‐3ʹ; β‐actin (F): 5ʹ‐CACCATTGGCAATGAGCGGTTC‐3ʹ, β‐actin (R): 5ʹ‐AGGTCTTTGCGGATGTCCACGT‐3ʹ. The TB Green® Premix Ex Taq™ II (RR820A; TaKaRa) was used to perform qPCR analysis. Gene expression relative to β‐actin was analyzed by the $2^{-∆∆C_{t}}$ method.

### 2.5 Western blot (WB) analysis

RIPA buffer (89901; Thermo Fisher Scientific) was used for protein extraction. The protein was separated by gel electrophoresis, followed by transfer onto polyvinylidene fluoride membranes. The membranes were blocked probed with RNF182 (1:1000, ab72533; Abcam), p65 (1:1000, 8242; CST), PDL1 (1:100, ab205921; Abcam), and β‐actin (1:200, ab115777; Abcam) overnight at 4°C, and then with IgG (HRP) (1:2000, ab6721; Abcam) at 22–25°C for 45 min. The enhanced chemiluminescence reagent (32209; Thermo Fisher Scientific) was used for color development, and the β‐actin served as the endogenous control for protein quantification.

### 2.6 Chromatin‐immunoprecipitation (ChIP)‐qPCR

The EZ‐Magna ChIP® G kit (17‐409; Sigma‐Aldrich, Merck KGaA) was used to analyze the binding between p65 and PDL1 promoter. In short, 1 × 10^7^ A549 or NCI‐H1975 cells were digested and collected for protein‐DNA crosslinking in formaldehyde. The crosslinking was terminated by glycine. Then, the cells were lysed and ultrasonicated for DNA truncation. The lysates were probed with the p65 antibody (1:100, 8242; CST) or rabbit IgG (1:100, ab171870; Abcam). Protein and DNA were decrosslinked by proteinase K treatment. DNA was collected and purified, in which the enrichment of PDL1 promoter fragments was analyzed by qPCR analysis.

### 2.7 Luciferase assay

To verify the p65's modulation on PDL1 promoter, the wild‐type (WT) sequence containing binding site of p65 with PDL1 promoter, or the mutant‐type (MUT) sequence, was inserted into the pGL3‐basic vector to construct PDL1‐WT and PDL1‐MUT luciferase reporter vectors. The vectors were administrated into 293T cells along with oe‐RNF182 and oe‐p65 using the Lipofectamine 3000 (L3000150; Thermo Fisher Scientific). Luciferase activity in cells was analyzed 48 h later using the dual luciferase examination kit (11402ES60; Yeasen Biotechnology).

### 2.8 Protein stability examination

The 293T cells were administrated with lentivirus vector‐carried oe‐RNF182 or oe‐NC. After 48 h, 100 μM cycloheximide (CHX; C7698; Sigma‐Aldrich) was added, and the cells were harvested at 0, 1, 2, and 4 h^15^ for WB analysis to analyze the p65 protein degradation.

### 2.9 Coimmunoprecipitation (Co‐IP)

HA‐p65, Flag‐Ub, and Myc‐RNF182 were transfected into 293T cells. After 48 h, the cells were treated with 20 μM MG132 (M7449; Sigma‐Aldrich) for 12 h^16^ and then harvested for Co‐IP following the instruction manual of the kit (AM001‐01; ACE Biotechnology). In brief, the cells were lysed and centrifuged at 13,000*g* for 10 min to discard the cell debris. The rProtein A/G MagPoly Beads were washed and incubated with the HA tag (1:30, ab236632; Abcam) overnight at 4°C. Afterward, the magnetic beads were collected to elute the protein. The eluted protein was detected by WB analysis. The antibodies used are as follows: HA tag (1:1000, ab236632; Abcam), Myc tag (1:1000, 2278; CST), and Flag (1:500, ab205606; Abcam).

### 2.10 Cell counting kit‐8 (CCK‐8) method

Following the instruction manual of the CCK‐8 kit (C0037, Beyotime Biotechnology Co., Ltd.), approximately 2000 cells were incubated in 96‐well plates. At 0, 24, 48, and 72 h of incubation, respectively, each well was filled with 10 μL CCK‐8 reagent, followed by 2 h of incubation.^17^ The optical density (OD)_450_ value was analyzed by a microplate reader.

### 2.11 Colony formation of cells

Approximately 1000 cells were seeded in each well of six‐well plates. After 14 days,^18^ the cells were washed, fixed, and stained by crystal violet. The cell colonies were counted under microscopy.

### 2.12 Transwell assays

Transwell chambers were used to analyze the migration and invasion (with additional Matrigel precoating) of LUAD cells. In short, approximately 4 × 10^4^ cells resuspended in serum‐free medium were filled into the apical chambers, and the basolateral chambers were added with 10% FBS‐containing medium. After 48 h,^19^ the cells migrated or invaded the lower membranes were fixed and stained with crystal violet for microscopy analysis.

### 2.13 Flow cytometry

Following the protocol of the apoptosis detection kit (V13242; Thermo Fisher Scientific), the cells were digested and counted. Approximately 5 × 10^4^ cells were resuspended and added to 96‐well plates, followed by incubation with Annexin V‐fluorescein isothiocyanate and propidium iodide reagent avoiding light exposure for 15 min. The cell apoptosis was then analyzed by the flow cytometer.

### 2.14 Cell cytotoxicity test

The cytotoxicity in LUAD cells was determined by a lactate dehydrogenase (LDH) kit (C0016; Beyotime). LUAD cells were used as target cells, while the CD8^+^ cytotoxic T cells (PCS‐800‐017; ATCC) were used as the effector cells. In short, 4 × 10^5^ LUAD cells and 6 × 10^5^ T cells were seeded into 96‐well plates. After 24 h of incubation,^20^ cells were centrifuged at 500*g*, and 120 μL cell culture supernatant was collected, which was incubated with 60 μL LDH avoiding light exposure for 30 min. The OD_490_ value was read by the microplate reader. The cytotoxicity in cells (%) = (LDH release in mixture of target and effector cells – LDH release in effector cells)/LDH release in target cells × 100.

### 2.15 Enzyme‐linked immunosorbent assay (ELISA)

After 24 h, the cocultured CD8^+^ T cells and LUAD cells were centrifuged at 500*g* for 5 min to collect supernatant, in which the interferon‐γ (IFN‐γ) and interleukin‐2 (IL‐2) concentrations were analyzed by the IFN‐γ (ab174443; Abcam) and IL‐2 (PI580; Beyotime) kits according to the standard curves based on the OD450 value of the standards.

### 2.16 Xenograft mouse models

Twenty C57BL/6J mice were acquired from Charles River Laboratory Animal Technology and divided in the oe‐NC, oe‐RNF182, oe‐RNF182 + oe‐NC, and oe‐RNF182 + oe‐p65 groups, *n* = 5 in each. A549 cells with corresponding transfections were injected into mice subcutaneously after resuspending in 100 μL phosphate‐buffered saline. After 1 week, the volume was evaluated weekly as follows: volume = 0.5 × length × width^2^. Four weeks later, the mice were killed by injecting excessive (150 mg/kg) 1% pentobarbital intraperitoneally and the tumors were collected and weighed. The usage of animals was ratified by the Animal Ethics Committee of the First Affiliated Hospital of Gannan Medical University (Approval No. LLSC‐2019032710).

### 2.17 Statistical analysis

Data were analyzed by Prism 8.02 (GraphPad). Measurement data were stated as the mean ± SD. The *t* test was applied to compare intergroup differences and one‐ or two‐way analysis of variance (ANOVA) along with Tukey's post‐hoc check was applied for difference analysis when over two groups are involved. Pearson's correlation analysis was used for gene correlation analysis. The correlation of RNF182 with the clinical feature of patients was analyzed by Fisher's exact test. Significant difference was set at *p* < .05.

## 3 RESULTS

### 3.1 RNF182 is poorly expressed in LUAD

**Figure 1:**
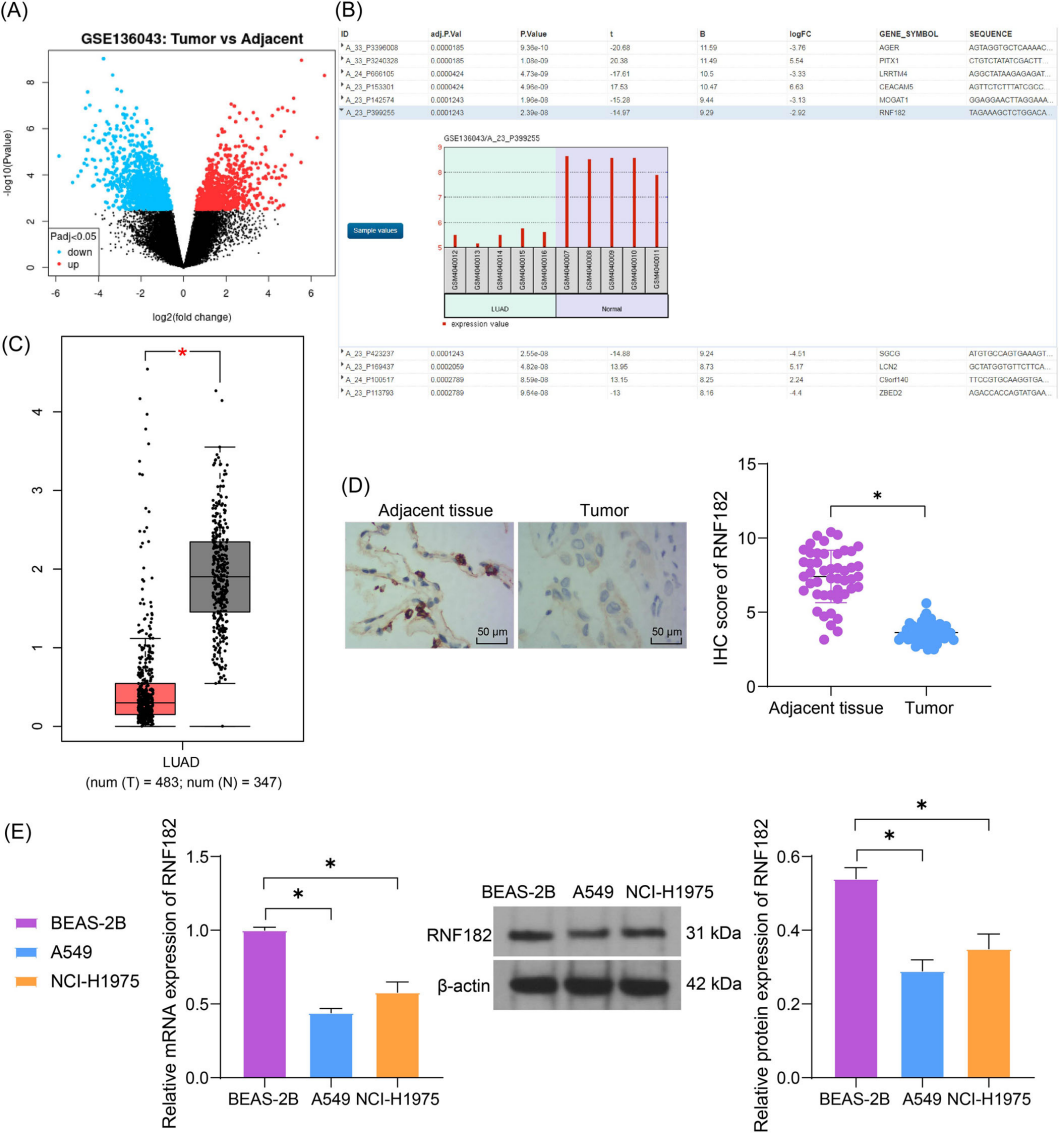
RNF182 is poorly expressed in LUAD. (A) Volcano plots for DEGs between LUAD tissues and nontumor lung tissues screened by analyzing the GSE136043 data set; (B) RNF182 is the only E3 ubiquitin ligase among the top 10 DEGs; (C) RNF182 expression in LUAD predicted in the GEPIA system; (D) IHC assay for RNF182 expression in the clinically collected LUAD tissues and adjacent normal tissues (*n* = 47); (E) RT‐qPCR and WB analysis for the mRNA and protein levels of RNF182 in the acquired LUAD cells (A549 and NCI‐H1975) and normal BEAS‐2B (*n* = 3). Differences were compared by the paired *t* test (D) or one‐way ANOVA (E). *p < .05. ANOVA, analysis of varience; DEG, differentially expressed gene; GEPIA, gene expression profiling interactive analysis; IHC, immunohistochemistry; LUAD, lung adenocarcinoma; RNF182, RING finger protein 182; RT‐qPCR, reverse transcription quantitative polymerase chain reaction; WB, western blot.

The GSE136043 data set containing gene expression data of five pairs of LUAD and non‐involved lung tissues were analyzed to identify DEGs in LUAD (Figure 1A). Among the DEGs, we found that RNF182 was the only E3 ubiquitin ligase (Figure 1B). Moreover, in the Gene Expression Profiling Interactive Analysis (GEPIA) database (http://gepia.cancer-pku.cn/index.html), we obtained that RNF182 is poorly expressed in LUAD (Figure 1C). This was validated by IHC assay that the RNF182 had a significantly decreased IHC score in the clinically collected LUAD tissues (Figure 1D). According to the average IHC score of RNF182, the patients were allocated into the high‐ and low‐RNF182 expression groups. As shown in Table 1, it was found that RNF182 had no correlation with the age and sex of patients, but had significant correlations with advanced TNM and clinical stages. Meanwhile, RT‐qPCR and WB assays identified decreased RNF182 expression in the LUAD cells (A549 and NCI‐H1975) compared to the normal BEAS‐2B cells (Figure 1E).

**Table 1:** Correlation of RNF182 expression with the clinical features of LUAD patients.

Clinic features	Sample size (*n* = 47)	RNF182 expression		*p* Value
		High (*n* = 22)	Low (*n* = 25)	
Age				
≥60	29	16	13	*p* > .05
<60	18	6	12	
Sex				
Male	31	17	14	*p* > .05
Female	16	5	11	
T stage				
T1	14	11	3	* *p* = .0092
T2–T4	33	11	22	
N stage				
N0	30	18	12	* *p* = .0318
N1–N2	17	4	13	
M stage				
M0	41	22	19	* *p* = .0234
M1	6	0	6	
Clinical stage				
Ⅰ–Ⅱ	34	20	14	* *p* = .0098
Ⅲ–Ⅳ	13	2	11	

*Note*: Fisher's exact test was used for correlation analysis.

Abbreviations: LUAD, lung adenocarcinoma; RNF182, RING finger protein 182.

* *p* < .05.

### 3.2 RNF182 restoration suppresses malignant properties of LUAD cells

**Figure 2:**
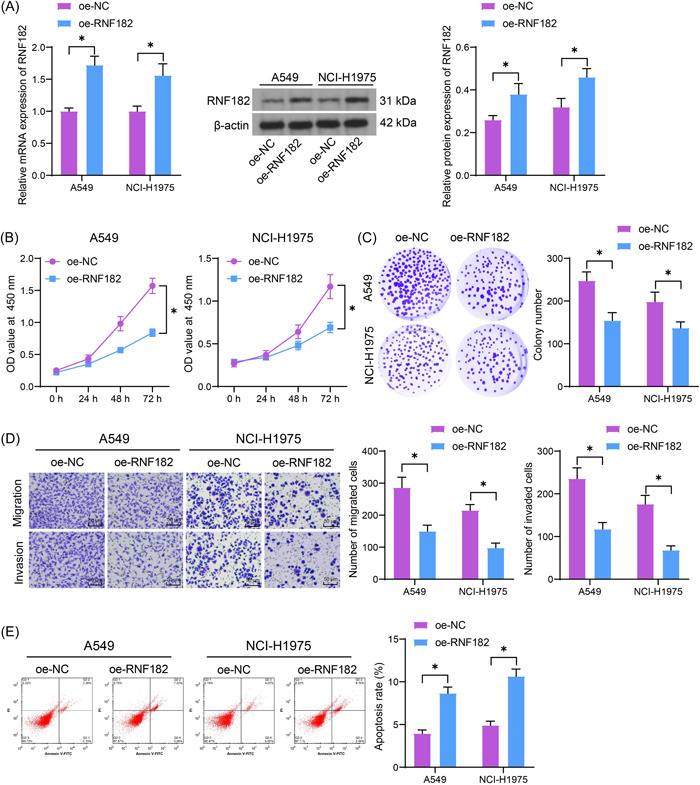
RNF182 restoration suppresses malignant phenotype of LUAD cells. (A) RT‐qPCR and WB analysis for the mRNA and protein levels of RNF182 in A549 and NCI‐H1975 cells after oe‐RNF182 transfection (*n* = 3); (B) CCK‐8 assay for the proliferation of the LUAD cells (*n* = 3); (C) number of the colonies formed by the LUAD cells (*n* = 3); (D) transwell assays for the migration and invasion capacities of the LUAD cells (*n* = 3); (E) flow cytometry for the apoptosis of the LUAD cells (*n* = 3). Differences were analyzed by the two‐way ANOVA (A–E). *p < .05. ANOVA, analysis of varience; CCK‐8, cell counting kit‐8; LUAD, lung adenocarcinoma; RNF182, RING finger protein 182; RT‐qPCR, reverse transcription quantitative polymerase chain reaction; WB, western blot.

The lentivirus‐carried oe‐RNF182 was transfected into A549 and NCI‐H1975 cells for gain‐of‐function assays. The successful RNF182 upregulation in cells was confirmed by RT‐qPCR and WB assays (Figure 2A). In the setting of RNF182 restoration, both the A549 and NCI‐H1975 cells showed decreased proliferation (Figure 2B) and colony formation (Figure 2C) abilities according to the CCK‐8 and colony formation assays. These cells had decreased migration and invasion capacities (Figure 2D). Moreover, the RNF182 upregulation increased the population of apoptotic A549 and NCI‐H1975 cells (Figure 2E).

### 3.3 RNF182 functions as an E3 ubiquitin ligase for p65 in LUAD

**Figure 3:**
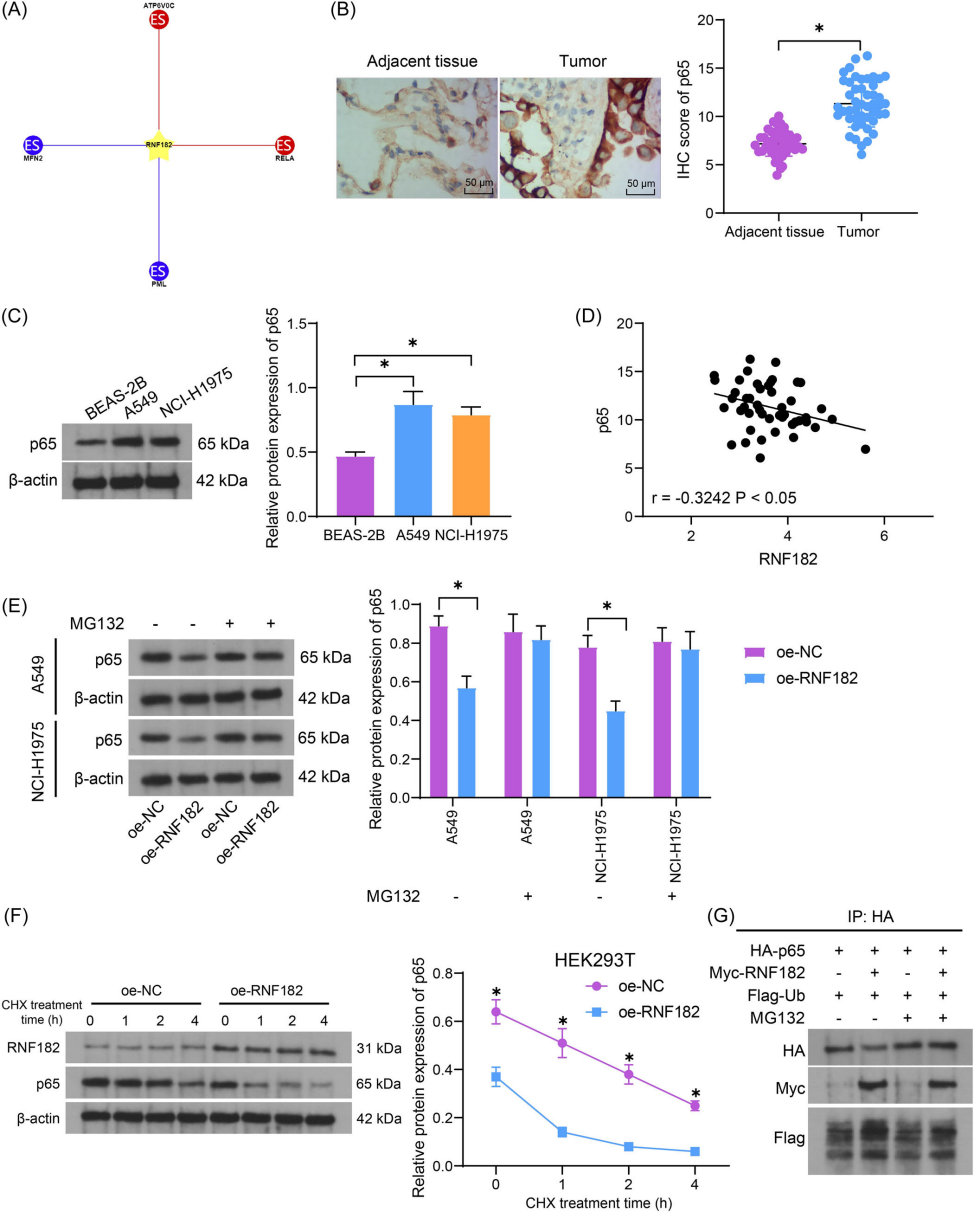
RNF182 functions as an E3 ubiquitin ligase for p65 in LUAD. (A) Target proteins of RNF182 as an E3 ubiquitin ligase predicted in the Ubibrowser 2.0 system; (B) IHC assay for the p65 expression in the clinically collected LUAD tissues and adjacent normal tissues (*n* = 47); (C) WB analysis for the protein level of p65 in the acquired LUAD cells (A549 and NCI‐H1975) and normal BEAS‐2B (*n* = 3); (D) an inverse correlation between RNF182 and p65 expression in the LUAD tissues (*n* = 47); (E) WB analysis for the protein level of p65 in oe‐RNF182‐transfected LUAD cells with or without MG132 treatment (*n* = 3); (F) WB analysis for the RNF182 and p65 protein levels in oe‐RNF182‐transfected‐293T cells at 0, 1, 2, 4 h after CHX treatment (*n* = 3); (G) 293T cells were transfected with HA‐p65, Myc‐RNF182, and Flag‐Ub and treated with MG132 and reacted with the HA antibody, and then the expression of HA, Myc, and Flag was analyzed by WB analysis (*n* = 3). Differences were compared by the paired *t* test (B), one‐way ANOVA (C), or two‐way ANOVA (E, F). In panel (D), Pearson's correlation analysis was performed. *p < .05. ANOVA, analysis of varience; IHC, immunohistochemistry; LUAD, lung adenocarcinoma; RNF182, RING finger protein 182; RT‐qPCR, reverse transcription quantitative polymerase chain reaction; WB, western blot.

Intriguingly, RNF182 has been reported as an E3 ubiquitin ligase regulating ubiquitination of p65,^8^ a subunit of the NF‐κB transcription factor involved in spanning oncogenic processes.^21^ In the Ubibrowser 2.0 (http://ubibrowser.bio-it.cn/ubibrowser_v3/home/index), RNF182 was predicted as an E3 ubiquitin ligase for p65 as well (Figure 3A). However, the interaction between RNF182 and p65 in LUAD has not been defined yet. Thereafter, we obtained via IHC and WB assays that p65 was abundantly expressed in LUAD tissues (Figure 3B) and cells (Figure 3C), respectively. The p65 expression in the collected LUAD tissues showed an inverse correlation with the RNF182 expression (Figure 3D). In cells transfected with oe‐RNF182, the p65 protein was substantially declined. However, the suppressive effect of RNF182 on p65 protein in the cells was blocked upon MG132 treatment (Figure 3E). Moreover, we treated the cells were CHX to evaluate the function of RNF185 in the protein stability of p65. The RNF182 overexpression reduced the half‐life of the endogenous p65 in the LUAD cells (Figure 3F). Afterward, we transfected HA‐P65, Myc‐RNF182, and Flag‐Ub into the 293T cells and treated the cells with MG132, followed by immunoprecipitation reaction using the HA antibody. In the absence of MG132 treatment, the Myc‐RNF182 transfection increased Flag‐Ub expression and decreased HA‐p65 expression in 293T cells. However, after MG132 treatment, the Myc‐RNF182 transfection increased Flag‐Ub expression but had no effect on HA‐p65 expression (Figure 3G).

### 3.4 Overexpression of p65 rescues the malignant properties of cancer cells

**Figure 4:**
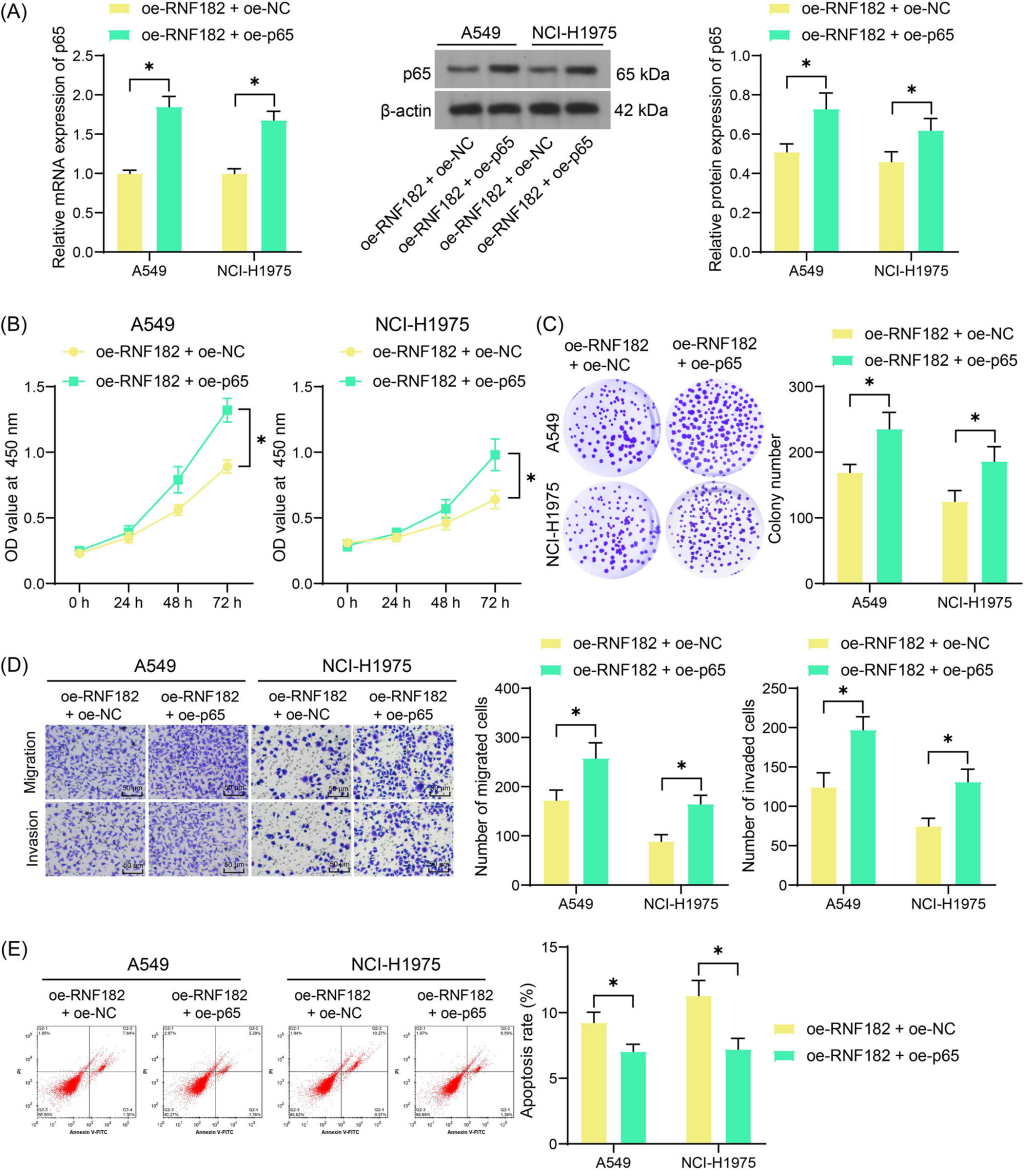
Overexpression of p65 rescues the malignant phenotype of cancer cells. (A) WB analysis for the protein level of p65 in A549 and NCI‐H1975 cells after oe‐p65 transfection (*n* = 3); (B) CCK‐8 assay for the proliferation of the LUAD cells (*n* = 3); (C) number of the colonies formed by the LUAD cells (*n* = 3); (D) transwell assays for the migration and invasion capacities of the LUAD cells (*n* = 3); (E) flow cytometry for the apoptosis of the LUAD cells (*n* = 3). Differences were analyzed by the two‐way ANOVA (A–E). *p < .05. ANOVA, analysis of varience; CCK‐8, cell counting kit‐8; LUAD, lung adenocarcinoma; RNF182, RING finger protein 182; WB, western blot.

To verify the interaction of RNF182 and p65 in LUAD progression, the A549 and NCI‐H1975 cells stably transfected with ‐transfected oe‐RNF182 were further administrated with lentivirus‐carried oe‐p65. The WB analysis confirmed the restoration of p65 protein in cancer cells (Figure 4A). Under this condition, the proliferation and colony formation of cells were substantially rescued (Figure 4B,C). Moreover, the oe‐p65 also recovered the mobility of LUAD cells inhibited by oe‐RNF182 (Figure 4D). Meanwhile, the apoptosis of both cells was suppressed by p65 overexpression (Figure 4E).

### 3.5 p65 activates the transcription of PDL1

**Figure 5:**
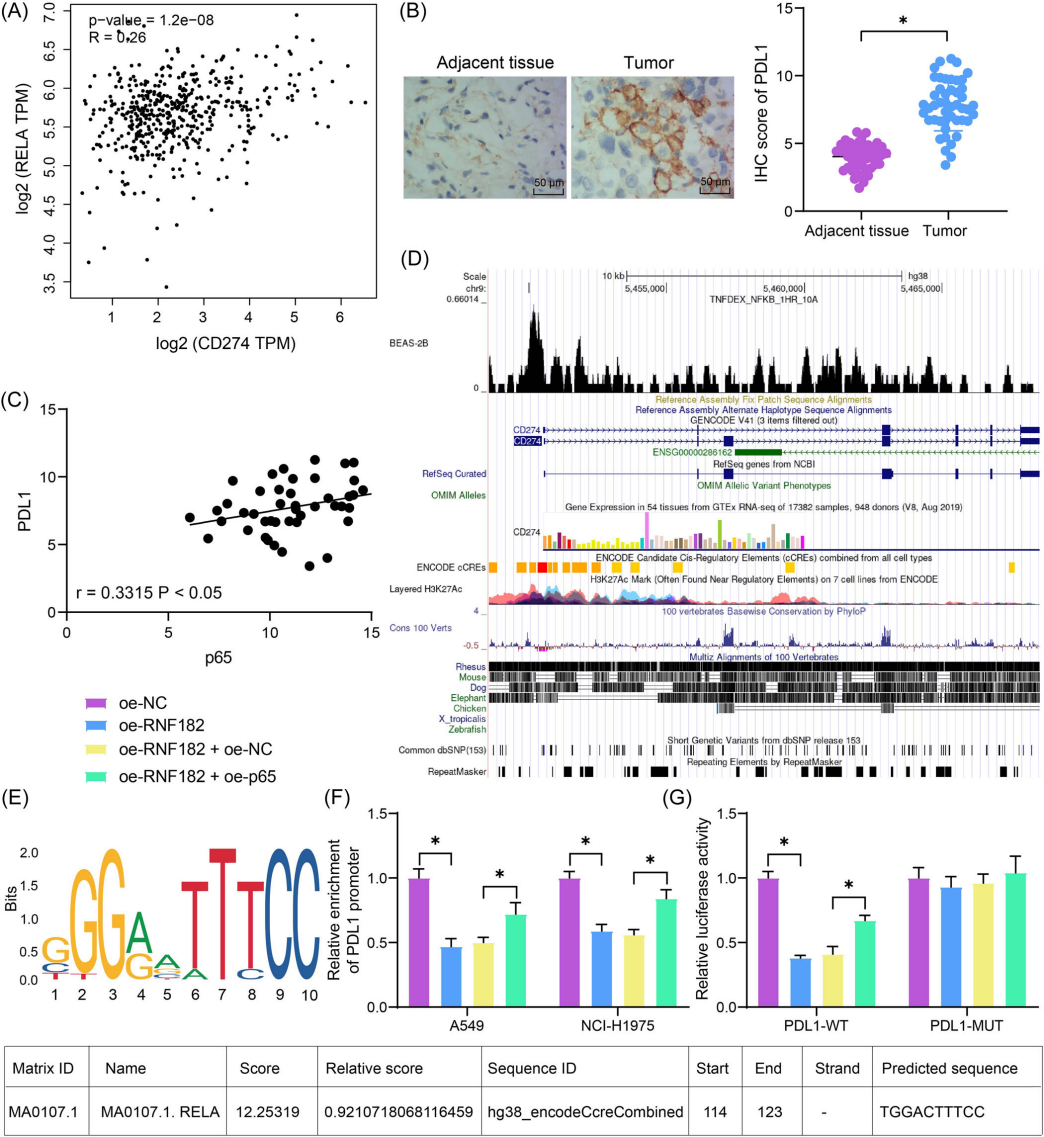
p65 activates the transcription of PDL1. (A) Correlation between p65 and PDL1 in LUAD predicted in the GEPIA system; (B) IHC assay for the PDL1 expression in the clinically collected LUAD tissues and adjacent normal tissues (*n* = 47); (C) a positive correlation of RNF182 and p65 expression in the LUAD tissues (*n* = 47); (D) binding peaks of p65 at PDL1 promoter region analyzed by the Cistrome Data Browser database; (E) binding sequence of p65 with PDL1 obtained from the JASPAR database; (F) abundance of PDL1 promoter fragments precipitated by the p65 antibody in LUAD cells after RNF182 or p65 overexpression (*n* = 3); (G) luciferase activity of the PDL1‐WT and PDL1‐MUT reporter vectors in 293T cells after RNF182 or p65 overexpression (*n* = 3). Differences were analyzed by the paired *t* test (B) or two‐way ANOVA (F, G). In panel (C), Pearson's correlation analysis was performed. *p < .05. ANOVA, analysis of varience; IHC, immunohistochemistry; LUAD, lung adenocarcinoma; PDL1, programmed death ligand 1; RNF182, RING finger protein 182.

p65 has been reported to bind to PDL1 promoter to stimulate the PDL1 transcription, leading to immunosuppression in tumors.^13^ Meanwhile, we queried the GEPIA system and obtained that the expression of p65 (RELA) showed a positive correlation with the PDL1 (CD274) expression (Figure 5A). The IHC assay confirmed an elevation of PDL1 expression in the collected LUAD cells (Figure 5B), which presented a positive correlation with p65 as well (Figure 5C). According to the Cistrome Data Browser (http://cistrome.org/db/#/), p65 is suggested to have significant binding peaks at the PDL1 promoter in A549 cells (Figure 5D). Thereafter, we predicted the binding sequence of p65 and PDL1 promoter in the JASPAR system (http://jaspar.genereg.net/) (Figure 5E). To validate the binding relationship, we performed the ChIP‐qPCR assay, by which we found that the abundance of PDL1 promoter fragments bound by p65 was reduced by RNF182 overexpression but increased by further p65 overexpression in the LUAD cells (Figure 5F). In the luciferase assay, the RNF182 overexpression suppressed the luciferase activity of the PDL1‐WT reporter vector but had little impact on the PDL1‐MUT vector; however, the p65 overexpression increased the activity of the PDL1‐WT luciferase reporter vector (Figure 5G).

### 3.6 The RNF182/p65 affects immunosuppression in LUAD cells by regulating PDL1

**Figure 6:**
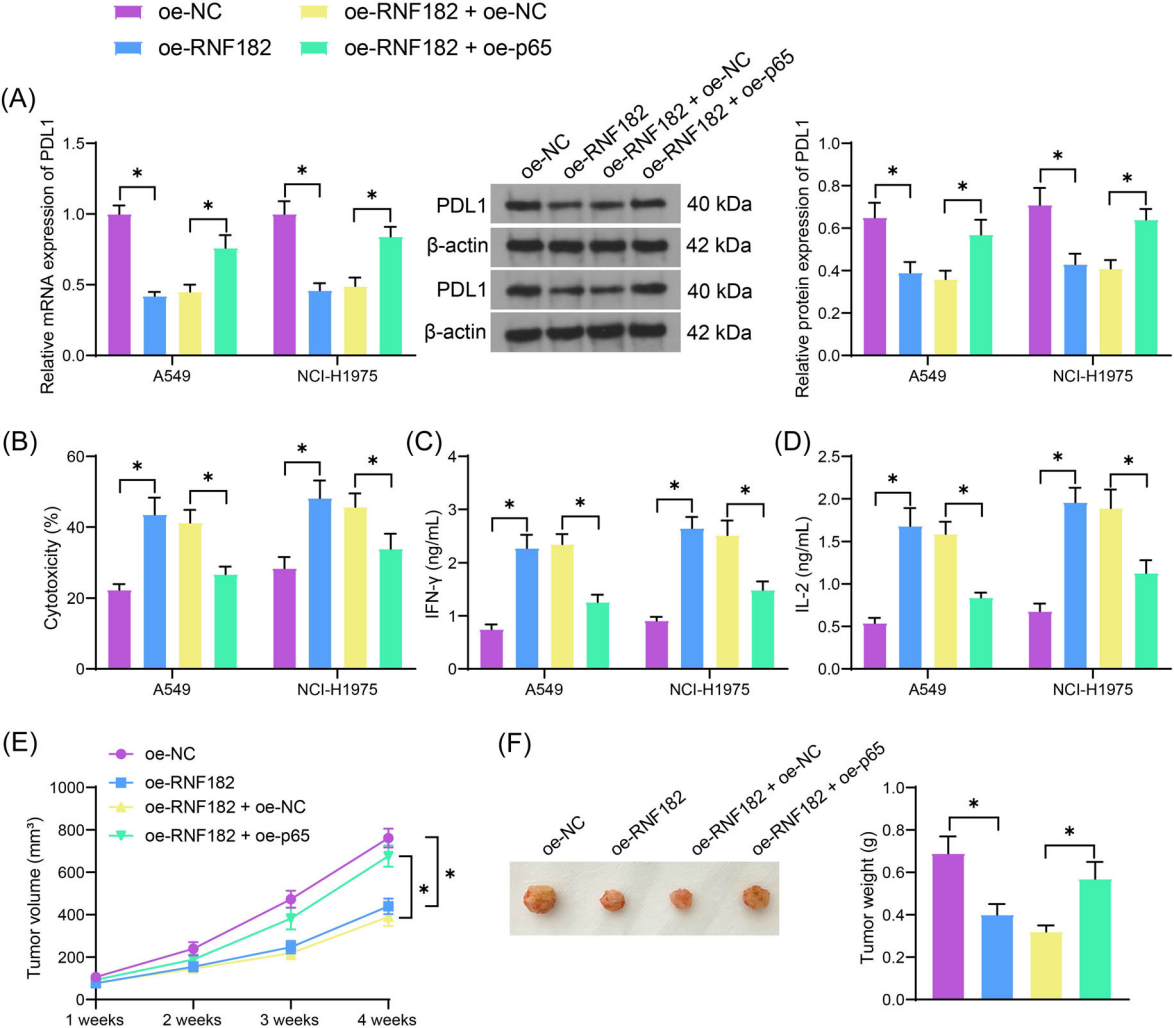
The RNF182/p65 affects immunosuppression in LUAD cells by regulating PDL1. (A) RT‐qPCR and WB analysis for the mRNA and protein levels of PDL1 in A549 and NCI‐H1975 cells (*n* = 3); (B) cytotoxicity in cancer cells after 24‐h coculture with CD8^+^ T cells analyzed by LDH release (*n* = 3); (C, D) ELISA kits for the concentrations of IFN‐γ (C) and IL‐2 (D) in the supernatant of cocultured cells (*n* = 3); (E) volume of xenograft tumors in mice on Week 1, 2, 3 and 4 after A549 cell implantation (*n* = 5); (F) weight of xenograft tumors in mice on Week 4 (*n* = 5). Differences were analyzed by the one‐way ANOVA (F) or two‐way ANOVA (A–E). *p < .05. ANOVA, analysis of varience; ELISA, nzyme‐linked immunosorbent assay; IFN‐γ, interferon‐γ; IL‐2, interleukin‐2; LDH, lactate dehydrogenase; LUAD, lung adenocarcinoma; PDL1, programmed death ligand 1; RNF182, RING finger protein 182; RT‐qPCR, reverse transcription quantitative polymerase chain reaction; WB, western blot.

Considering the PDL1 is a key immune checkpoint responsible for immunosuppression, we, therefore, focused on the functions of the RNF182/p65 axis in the immune activity in LUAD cells. First, the PDL1 expression in cells was detected. The RT‐qPCR identified that the PDL1 expression in the A549 and NCI‐H1975 cells was blocked by oe‐RNF182 but rescued by oe‐p65 (Figure 6A). In the coculture system of LUAD cells with CD8^+^ cytotoxic T cells, the cytotoxicity in cancer cells was analyzed by LDH release. Importantly, the LDH release in both cell lines was increased by RNF182 overexpression but suppressed by p65 restoration (Figure 6B). Moreover, the ELISA results showed that the RNF182 overexpression resulted in an increase in the contents of Th1 cytokines IFN‐γ and IL‐2 in the supernatant of LUAD cells. However, the concentrations of these cytokines were decreased by p65 upregulation (Figure 6C,D). In the xenograft mouse models, the RNF182 overexpression in A549 cells led to retarded growth rate and reduced weight in mice. Likewise, further upregulation of p65 in cells promoted the tumor growth rate and the tumor weight (Figure 6E,F).

## 4 DISCUSSION

Lung cancer remains a huge global health burden, and ongoing studies aim to identify more key pathogenic molecules to develop more potential strategies for cancer screening and treatment.^22^, ^23^ This work reports that the restoration of RNF182 in LUAD results in increased immune activity and tumor suppression, which is ascribed to its regulation on the ubiquitination and degradation of p65 protein and the subsequent transcriptional inactivation of PDL1.

Studies concerning the tumor‐affecting E3 ubiquitin ligases abound. For example, a recent work by Li et al. reports that the Makorin RNF protein 3 catalyzes the ubiquitination of poly(A) binding protein cytoplasmic 1 for degradation, which consequently blocks the proliferation of lung cancer cells.^24^ By contrast, the ligase protein HRD1 augments the growth and tumorigenicity activity of lung cancer cells by inducing sirtuin 2 ubiquitination and degradation.^25^ RNF126, another member of the RNF protein family, has been found to promote the malignant properties of human bladder cancer cells by mediating the ubiquitination of the tumor suppressor protein phosphatase and tensin homolog.^26^

In this work, RNF182 was obtained as the only E3 ubiquitin ligase among the top 10 DEGs between LUAD and nontumor tissues in the GSE136043 data set. RNF182 is among the least studied member of the RNF183 family with the current findings mainly focusing on its role in neural impairments.^7^ The correlation of RNF182 with cancer development, to the best of we know, has barely been investigated. Here in this work, we validated decreased RNF182 expression in LUAD samples and figured out the link of low RNF182 expression to the advanced tumor stage of LUAD patients. Coordinating with the functional assay results that the RNF182 restoration blocked the proliferation, mobility, resistance to apoptosis, and tumorigenic activity of LUAD cells, we report that RNF182 plays a tumor‐suppressive effect on LUAD. This evidence indicates that RNF182 may serve as a favorable prognostic marker in patients with LUAD.

As mentioned above, RNF182 has been reported to modulate the ubiquitination of p65 protein.^8^ Similarly, a recent report by Liu et al. demonstrates that RNF182 mediates p65 ubiquitination and degradation to block the binding of p65 with solute carrier family 7 member 11, therefore, participating in the regulation of ferroptosis and tumor elimination of hepatocellular carcinoma.^27^ As a well‐defined oncogenic transcription factor, the ubiquitination and protein degradation of p65 has been found to suppress malignant properties of lung cancer cells.^28^ Subsequently, we identified that the RNF182 showed an inverse correlation with the p65 protein level in LUAD tissues and confirmed the regulation of RNF182 on p65 ubiquitination in LUAD cells. Considering that the further p65 upregulation blocked the effects of RNF182 overexpression on LUAD cells, we opined that the p65 degradation is implicated in the tumor‐suppressive roles of RNF182.

The NF‐κB p65 has been defined as a transcriptional activator of PDL1.^29^, ^30^ Cancer cell‐expressed PDL1 can function as an “adaptive immune mechanism” to evade the antitumor immune responses.^31^ As an immune checkpoint receptor, the primary mechanism of PDL1 in immune evasion is to interact with programmed cell death 1 (PD‐1) to induce exhaustion of CD8^+^ T cells and inhibit their antitumor efficacy.^32^, ^33^ However, the effective rate of anti‐PDL1 antagonists for PDL1‐positive metastatic lung cancer treatment was reportedly only 40%–50% due to the complex tumor microenvironment.^34^ By the way, in addition to activating PDL1 transcription, p65 has been found to induce immune suppression through other mechanisms. For instance, it has been reported to activate immunosuppression‐associated cytokines such as colony‐stimulating factor 1 and C‐X‐C motif chemokine ligand 1, consequently leading to M2 skewing of tumor‐associated macrophages to induce immune evasion and tumor metastasis.^35^, ^36^ Additionally, p65 has been demonstrated to be necessary for the upregulation of C‐C motif chemokine ligand 20, which recruited the immunosuppressive regulatory T cells to induce immune evasion.^37^ Not surprisingly, in lung cancer, p65 has been found to affect the immune escape by modulating the PDL1 transcription as well.^13^, ^38^, ^39^ Here in this work, we validated the binding between PDL1 and p65 in LUAD cell lines. More importantly, the RNF182 overexpression blocked the binding relationship and subsequently led to a decline in PDL1 expression. Moreover, we found that the RNF182 overexpression increased the LDH release by LUAD cells and the IFN‐γ and IL‐2 production in the coculture system of LUAD cells and cytotoxic T cells. IFN‐γ and IL‐2 are key effector molecules linked to type‐1 pro‐inflammatory immune responses mediated by the CD8^+^ T cells directed towards cancer cells.^40^ Collectively, this body of evidence indicates that RNF182 has the capacity to reduce immune evasion and malignant growth of LUAD cells.

## 5 CONCLUSION

In conclusion, this work defines a novel tumor‐suppressive E3 ubiquitin ligase RNF182 which induces ubiquitination and degradation of p65 and consequently suppresses the transcription of PDL1, therefore, alleviating immunosuppression and cancer development. These findings may offer new ideas that RNF182 may be used as a favorable prognostic marker potentially correlated with better outcome of patients. Specific upregulation of RNF182 may help reduce immune escape and limit tumor growth in LUAD, though much more preclinical research is required to validate our findings.

## AUTHOR CONTRIBUTIONS

**Xingdu Zeng**: Conceptualization; data curation; methodology; supervision; validation; visualization; writing—original draft; writing—review and editing. **Xiaoyuan Tang**: Data curation; software; supervision; validation; visualization; writing—review and editing. **Xingxiang Chen**: Data curation; investigation; methodology; validation; writing—original draft; writing—review and editing. **Huilan Wen**: Data curation; formal analysis; funding acquisition; validation; visualization; writing—review and editing.

## CONFLICT OF INTEREST STATEMENT

The authors declare no conflict of interest.

## Data Availability

All data generated or analyzed during this study are included in this published article.
